# Intracardiac ultrasound‐guided left bundle branch pacing in a bradycardia patient

**DOI:** 10.1002/ccr3.2798

**Published:** 2020-03-11

**Authors:** Jiefang Zhang, Yaxun Sun, Zuwen Zhang, Chenyang Jiang, Guosheng Fu

**Affiliations:** ^1^ Department of Cardiology Sir Run Run Shaw Hospital Zhejiang University of Medicine Hangzhou China

**Keywords:** interventricular septum, intracardiac ultrasound, left bundle branch pacing

## Abstract

Left bundle branch pacing was associated with narrower QRS in patients with pacemaker indications. LBBP guided by ICE improves the accuracy of LBBP, facilitates the lead localization, minimizes complications, and above all reduces radiation exposure time.

## Graphical abstract

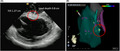

## 1 INTRODUCTION

Left bundle branch pacing (LBBP) was associated with narrower QRS width and lower pacing threshold in patients with pacemaker implantation indications. The application of intracardiac ultrasound (ICE) might improve the success rate of pacing in the left bundle branch area, facilitate the lead localization, and minimize complications including perforation and tricuspid regurgitation. In our case, LBBP guided by ICE significantly reduced radiation exposure time and do not increase the operation time.

Left bundle branch pacing (LBBP) has recently been demonstrated as feasible and clinical beneficial as a novel pacing technique and characterized by lower and stable pacing threshold, relatively narrower QRS duration due to fast left ventricular activation.^1^, ^2^ Precise identification of pacing site is critical for this technology. However, the LBBP technique is still a challenge for many operators, especially the judgement of the depth and angle of the lead. At present, the depth of lead implantation depends on the operator's experience and intracardiac ECG characteristics. We present a case of ICE‐guided LBBP in a bradycardia patient, which the depth and angle of the lead could be directly observed in the implantation procedure. This approach improved accuracy of the lead placement and reduced radiation exposure.

## 2 CASE PRESENTATION

**Figure 1:**
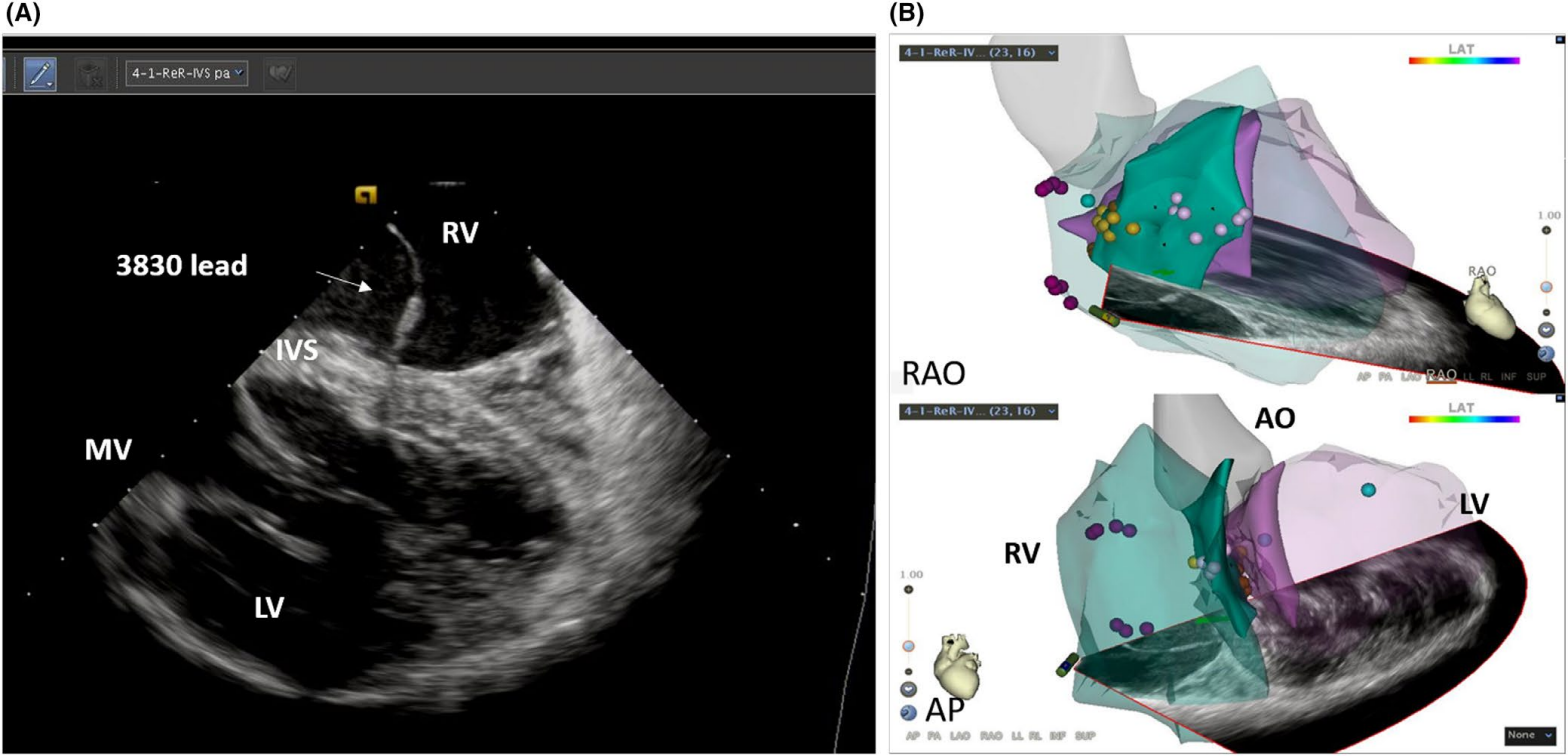
The initial site of the lead and the relationship of the interventricular septum in ICE. A, Intracardiac ultrasonic section showed the left and right ventricles and IVS, the 315 sheath and the initial site of the lead (small white arrow). B, The RAO and AP view showed the IVS model established by the ultrasonic catheter, the position of the ultrasonic catheter, and ultrasonic section. The ultrasonic catheter is located in the right ventricle and the ultrasonic section points to the septum. Abbreviations: AO, aorta; AP, anteroposterior; IVS, interventricular septum; LV, left ventricle; MV, mitral valve; RAO, right anterior oblique; RV, right ventricle

**Figure 2:**
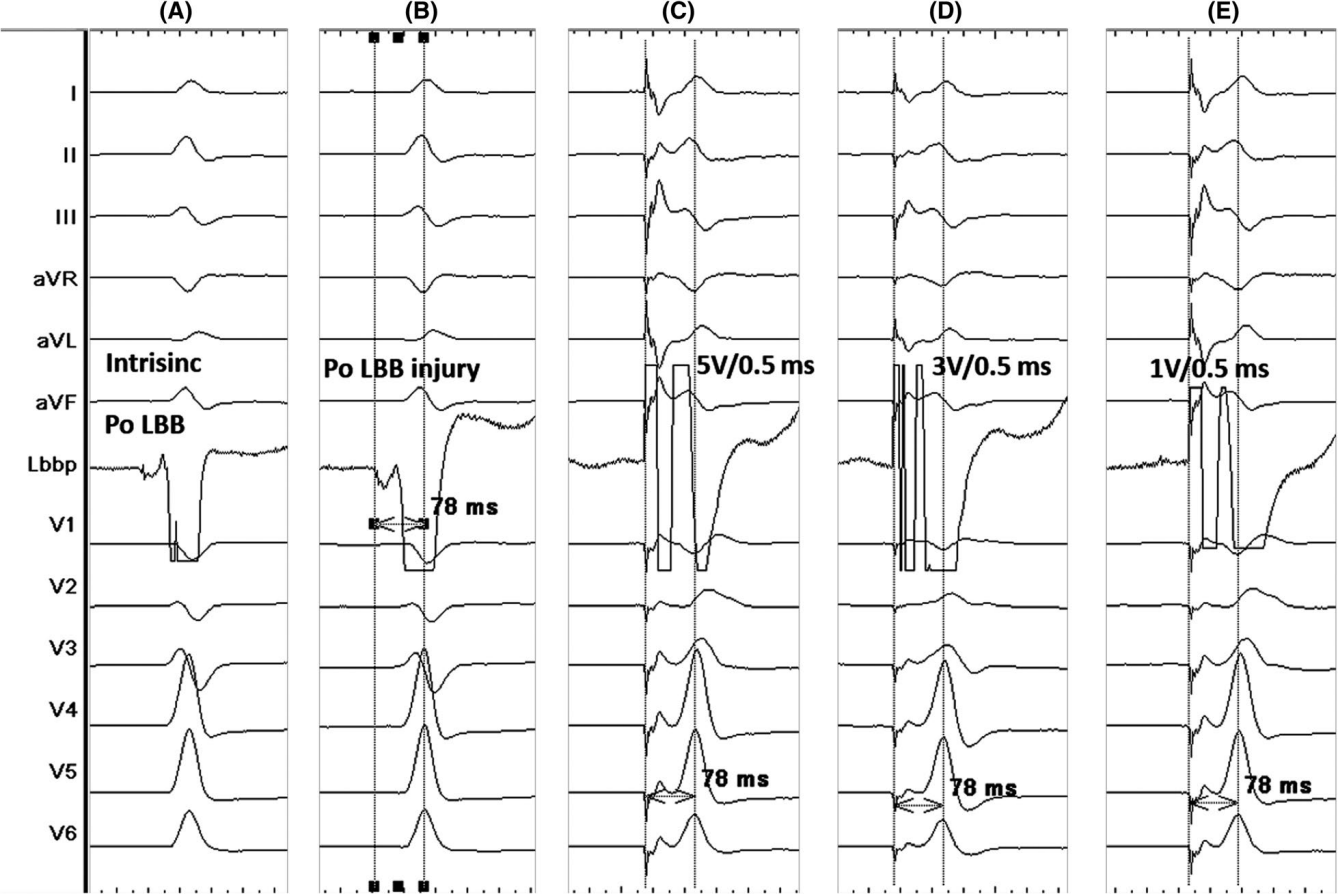
12‐lead ECG and lead recorded electrogram during implantation. A, LBB potential. B, LBB potential injury. C, Pacing in 5 V/0.5 ms (unipolar), Stim‐LVAT = 78 ms; D, Pacing in 3 V/0.5 ms (unipolar), Stim‐LVAT = 78 ms; E, Pacing in 1 V/0.5 ms (unipolar), Stim‐LVAT = 78 ms. Indicate LBB capture with the shortest and constant Stim‐LVAT of 78 ms. Po LBB = LBB potential

**Figure 3:**
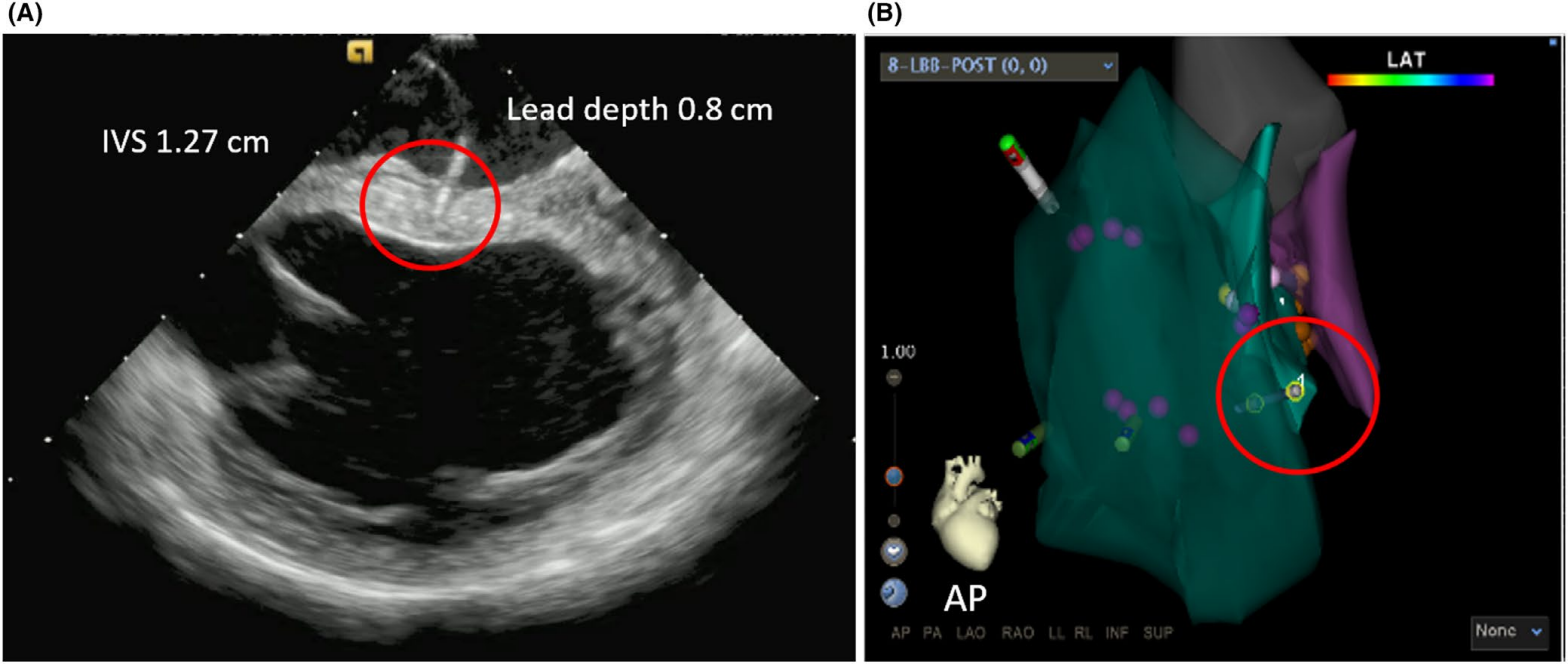
The process of pacing lead implantation in real time of CARTO 3 mapping system. A, The depth and angle of the lead screwing into IVS can be seen in ICE. The measured ventricular septum thickness was 1.27 cm, the lead helix screw into the midlevel of ventricular septal with depth of 0.8 cm. B, AP view showed the ventricular septal model established by the ultrasonic catheter, which is located in the right ventricle. The lead located in the middle of the interventricular septum. Abbreviations: AP, anteroposterior; IVS, interventricular septum

A 61‐year‐old male patient was admitted of bradycardia underwent a permanent double chamber pacemaker implantation. An intracardiac ultrasound proprietary catheter (CARTOSOUND; Biosense Webster) was advanced to the right atrium through femoral vein access. Meanwhile, the CARTO 3 mapping system (Biosense Webster) was applied to reconstruct the ventricle and interventricular septum models. Then, the 3830 lead (Select‐Secure™ lead, Medtronic) was connected to the CARTO 3 mapping system and advanced into right ventricle (RV) through sheath 315HIS (Medtronic) under ICE real‐time imaging guidance. During the operation, the sector of ICE was adjusted to clearly display both sides of the ventricular septum, and the position of the lead was observed directly (Figure 1). The sheath was adjusted to the upper region of the IVS under ICE real‐time imaging guidance. At this site, the paced QRS morphology usually demonstrates a “w” pattern in lead V1. The sheath was rotated counterclockwise to maintain the orientation of the lead tip perpendicular to the septal surface. During the time, EMG morphology changes of V1 lead, LBB potential, lead depth, and pacing impedance were closely monitored. Confirmation of the LBB capture was done according to Vijayaraman P and Huang.^1^, ^3^ Including: Paced morphology of right bundle branch block pattern; Identification of the LBB potential; Stim‐LVAT (stimulus to left ventricular activation time, defined as the interval from the pacing stimulus to the QRS upstroke in V5/V6) that shortens abruptly with increasing output or remains shortest and constant both at low and high outputs. Then, the pacing and EMG parameters of LBBP were measured. In this case, Stim‐LVAT was 68 ms, and paced QRS was 115 ms (Figure 2). The sensed R‐wave amplitude was 7.1 mV, capture threshold was 0.7 V/0.4 ms, and lead impedance was 726 Ω. Ultrasonic images showed the lead tip was perpendicular to the interventricular septum, with depth of 8 mm (Figure 3). LBBP was successfully achieved, with a radioactive exposure time of 1.5 minutes, and total operation time 76 minutes.

## 3 DISCUSSION

The main technological difficulty of LBBP lies in the positioning of the pacing lead in the LBB area. At present, the positioning of the pacing lead mainly depends on X‐ray images and intracardiac electrocardiogram, together with the experience of operators. Visible and reliable intraoperative imaging methods still lack. Intracardiac three‐dimensional ultrasound can reconstruct the three‐dimensional model of the heart by outlining the structure of the cardiac cavity seen by the two‐dimensional sector. ICE is widely used in electrophysiological intervention.^4^ It has been reported that ICE was used in animal experiments to perform His pacing.^5^ ICE can show the position of pacing lead in real time and can clearly show the depth of lead screwed into myocardium. At present, conduction bundle pacing is widely carried out, especially LBBP. Active pacing leads often need to be deeply screwed into the LBB area, and the angle needs to be adjusted to increase the success rate of capturing the LBB. Conventional x‐rays do not determine the angle and depth of the lead into the myocardium, making procedure more difficulty and risky (ventricular septum perforation). Intracardiac ultrasound‐guided LBBP can prevent the leads from penetrating the ventricular septum into the left ventricle, improve the success rate, and safety of implantation.

## CONFLICT OF INTEREST

The authors declared no potential conflicts of interest with respect to the research, authorship, and/or publication of this article.

## AUTHOR CONTRIBUTIONS

ZJ: served as a surgeon, wrote the original draft and collected the data. SY: reviewed the manuscript. ZZ: served as an electrophysiological technician and contributed software. JC: served as a surgeon and edited the manuscript. FG: devised the methodology and edited the manuscript.
